# A feasibility study of training in a local community aimed upon health promotion with special emphasis on musculoskeletal health effects

**DOI:** 10.12688/f1000research.73698.1

**Published:** 2022-01-17

**Authors:** Rizky Suganda Prawiradilaga, Magnus Bendtsen, Simon Esrup, Niklas Rye Jørgensen, Fajar Awalia Yulianto, Eva Wulff Helge

**Affiliations:** 1 Department of Biochemistry, Nutrition and Biomolecular. Faculty of Medicine, Universitas Islam Bandung, Bandung, Jawa Barat, 40116, Indonesia; 2 Department of Nutrition, Exercise and Sports, University of Copenhagen, Copenhagen N, 2200, Denmark; 3 Department of Clinical Biochemistry, Rigshospitalet Glostrup, Glostrup, 2600, Denmark; 4 Odense Patient data Explorative Network (OPEN), Odense University Hospital/Institute of Clinical Research, University of Southern Denmark, Odense, 50000, Denmark; 5 Department of Public Health, Faculty of Medicine, Universitas Islam Bandung, Bandung, Jawa Barat, 40116, Indonesia

**Keywords:** bone mineral density, bone turnover marker, osteoporosis, high-impact, odd-impact, resistance exercise, balance training, multimodal training

## Abstract

**Background:** To minimize fracture risk, multimodal training regimens are recommended. However, their effectiveness in community settings remains uncertain. This study evaluated the feasibility of 19-weeks of multimodal training in a local community center with emphasis on musculoskeletal health in postmenopausal women.

**Methods:** In a controlled trial, 28 postmenopausal women (53-68-years-old) were assigned to a multimodal training group (MMT, n=15) or a control group (CON, n=13). The training consisted of high- and odd-impact, resistance and balance-coordination training 1-2 hours weekly. The outcomes were attendance rate, regional and total bone mineral density (BMD), bone mineral content (BMC), bone turnover markers (BTM), body composition, functional muscle strength and power, and dynamic balance. All were determined at baseline and after 19 weeks of training. BTM was assessed after three weeks.

**Results:** Overall, 22(79%) participants (MMT, n=9; CON, n=13) completed the study, and the mean attendance rate for MMT was 65.5% of the maximum sessions (2) offered. Only right trochanter BMD increased (p<0.05) by 1.0±1.1% in MMT, which was higher(p<0.05) than CON. While whole-body BMC was not changed at 19 weeks from baseline in MMT, it decreased (p<0.05) in CON resulting in a significant difference (p<0.05) in whole-body BMC delta values between the two groups. Compared to baseline, body fat percentage(%BF), fat mass(FM), and visceral adipose tissue (VAT)-mass and -volume were decreased (p<0.01) in MMT, and were larger (p<0.05) than CON. No significant changes were observed in BTM, muscle strength and power, and dynamic balance after 19 weeks.

**Conclusions:** Nineteen weeks of multimodal training 1-2 hours per week in a local community had a health-enhancing effect on %BF, FM, and VAT, whereas the musculoskeletal health impact was modest. We hypothesize that the reason might be too low training volume and frequency and supposedly too low musculoskeletal training intensity for some participants.

**Registration:** ClinicalTrials.gov NCT05164679 (21/12/2021).

## Introduction

Osteoporosis is a growing public health concern. The disease is characterized by impaired bone strength due to the reduction of bone mass and impairment of the micro-architecture of the bone, which increase the risk of bone fracture. ^1^ It is estimated that 200 million females are affected worldwide and causes more than 8.9 million fractures per year. ^2^ Falls in osteoporotic patients are linked to high morbidity and mortality. ^3^ Therefore, fall prevention strategies while improving the bone strength aimed at the high-risk population are needed. ^4^

One way to prevent osteoporosis can be through engaging in evidence-based training to improve bone mineral density (BMD) and reduce age-related bone loss, ^5^ such as high-impact and resistance training. Furthermore, balance and coordination training can minimize the risk of falls and fractures, which is relevant to the high-risk population. ^4^ Studies have shown that high-impact exercises, which produce high vertical ground reaction forces with a high force development rate, ^6^ are beneficial for bone structure and mineralization in children, ^7^ adults, ^8^ and the elderly. ^9^ In a previous study of the acute osteogenic response to high-impact jumping in postmenopausal women, ^10^ stimulation of bone formation without any increase in bone resorption after jumping was reported. Moreover, the latter study showed a dose-response relationship between vertical as well as combined three-axes ground reaction forces (GRF) and the acute procollagen type I amino-terminal propeptide (P1NP)-response after countermovement jumps.

In addition, resistance exercise, which induces various muscle loads applied to the bone, ^11^ is reported to be a relevant osteogenic training modality for maintaining or increasing bone density and mass in older people. ^12^ Besides, to prevent falls, balance and coordination exercises are crucial to be included as they improve static and dynamic balance. ^13^ Therefore, it is hypothesized in the present study that evidence-based multimodal training as health promotion will have an osteogenic effect on bone mass and bone strength that may counteract the normal age-related bone loss in postmenopausal women and improve the dynamic balance as well as muscle strength and power, which may prevent falls and reduce the fracture risk.

Although the association between exercise training, bone loss, and falls prevention in older adults appear to be established in intervention studies, ^14^ only a few studies have examined the effects of combined training on musculoskeletal health in postmenopausal women, ^15^^–^^17^ and the results are varied. Therefore, there is still a need for safe, reliable, affordable, and evidence-based training programs that can be introduced, embraced, and sustained in high-risk populations, such as postmenopausal women.

The present study was performed in a local community setting. The aim was to investigate the feasibility and the health-promoting musculoskeletal effects of 19wk multimodal training (high- and odd-impact, resistance, balance, and coordination training) for postmenopausal women. The primary outcomes were bone mass and BTM, while body composition, dynamic balance, muscle strength and power were the secondary outcomes.

## Methods

### Study design

The multimodal training was offered to postmenopausal healthy women in a local community. The outcomes for the training group (MMT) were compared with a sedentary control group (CON). Training was offered twice weekly for one hour, and the average attendance rate of MMT participants over the 19wk had to be > 1 hour weekly. To collect baseline (BASE) data, all participants showed up in the laboratory on three occasions prior to the intervention (a description of the specific methods used is given in later sections). On the first visit, resting blood samples were collected to evaluate the concentration of bone turnover markers (BTM), and body weight, height, body composition including BMD and bone mass were measured. On the second visit, a dynamic balance test (four square step test) and a functional muscle strength and power test (jump-and-reach test) were performed. On the third visit, the aerobic capacity was evaluated by assessment of VO2-max. After three weeks (3wk) of training, resting blood samples were additionally collected. After 19wk of training, all assessments were repeated and compared with baseline and MMT and CON were compared.

Blood sample collection, DXA-scanning, and VO2-max tests were carried out at the Department of Nutrition, Exercise and Sports, University of Copenhagen, Denmark. The testing of vertical jump height, as well as dynamic balance, were performed at the health promotion training initiative in the Copenhagen area, Denmark. The blood samples were analyzed at the Department of Clinical Biochemistry, Rigshospitalet Glostrup Hospital, Denmark.

To ensure transparency of the study protocol, the trial was registered retrospectively at ClinicalTrials.gov and published on December 21 ^st^, 2021 (NCT05164679). As registration at ClinicalTrials.gov has until recently not been a part of the general research policy in our team, the trial was registered after completion of the study.

### Participants

Healthy, sedentary postmenopausal women aged below 70 years were eligible to participate in the present study. Inclusion criteria were non-smoker and body mass index (BMI) <30 kg/m ^2^. Exclusion criteria were: T-score < -3 SD in the lumbar spine or hip; Z-score > 1.5 SD (high age-related BMD); use of hormone therapy, medical treatment, or supplements that affect bone metabolism; previous or current medical condition affecting bone health; engagement in regular and systematic weight-bearing training or strength training during the preceding two years.

**Figure 1.**
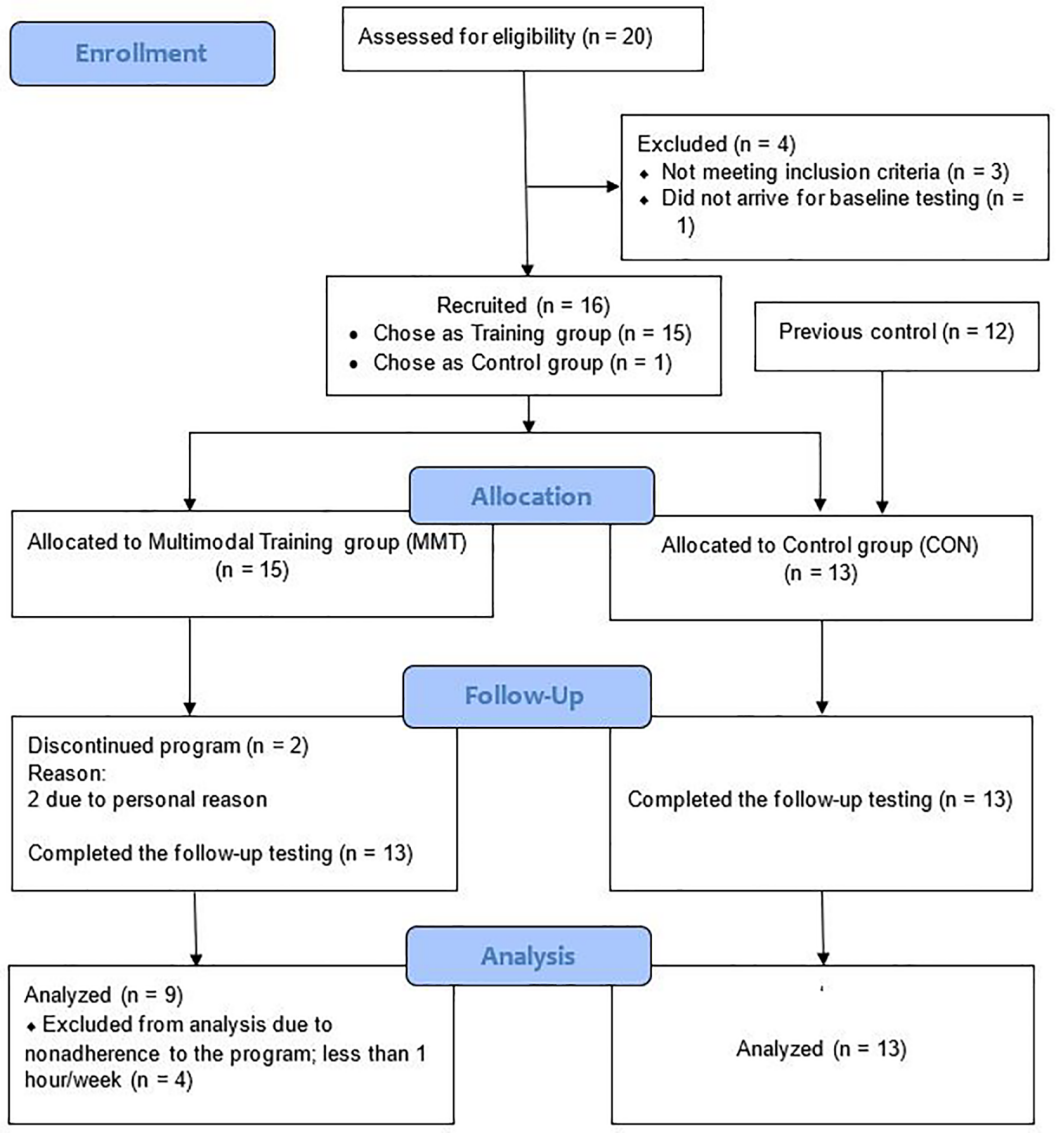
CONSORT flow diagram.

Initially, twenty women were recruited to the training via advertisements online and an in a local newspaper, of which 19 showed up for pre-testing ( Figure 1). After a medical examination, one participant was excluded due to low BMD (T-score < −3 SD), and two were excluded due to high BMI (≥30 kg/m ^2^). In addition, one woman refrained from participating in the training, and thus, 15 participants were recruited to MMT. During the study, two participants in MMT dropped out due to personal reasons and at the end of the study four participants were excluded in the analyses as their average attendance to the program was less than one hour per week. CON consisted of 13 age-matched sedentary postmenopausal women (12 from a previous study plus the woman who refrained from participating in the training), and therefore 9 MMT and 13 CON was finally included in the data analyses. Participants' baseline characteristics, including maximum oxygen consumption (VO2-max), are shown in Table 1.

Every participant was fully informed in writing and verbally before giving her written consent to the procedures and potential discomfort associated with the study. The study was conducted in accordance with the Declaration of Helsinki and approved by the local ethics committee of the Capital Region of Denmark, H-18044190.

### Training program

The present feasibility study evaluated the osteogenic impact of a training concept already offered by a health promotion initiative, “Knoglestærk” (“Bone strong”), in a local community.

According to the initiative, the training was offered as “evidence-based bone training” aimed upon enhancing musculoskeletal health. It was carried out 2 × 60 minutes weekly. The training concept included: 1) High- and odd-impact exercise including multi-directional games; 2) Progressive resistance training; 3) Balance and coordination training. The participants were engaged in intermittent gymnastics and small game sessions aimed at imposing osteogenic and diverse strain on the skeleton, mainly in the legs and arms. Thus, the training included various jumping exercises (e.g., counter-movement jumps, jumps from the floor up onto a bench, and from a bench down to the floor), quick walking up and down on gymnastics equipment, sprinting over short distances in different directions (in small games form). In addition, resistance exercises with elastic power bands, heavy exercise balls (medicine balls), and sandbags of 5-7 kg were applied. The training was performed intermittent and varied to ensure that the bones would not be desensitized, as it is observed in repetitive endurance training.

### BMD and body composition

To test the BMD exclusion criteria at baseline as well as evaluate bone adaptation in proximal femur (PF), lumbar spine (LS), and whole-body (WB) after 19wk of training, BMD (g/cm ^2^) were assessed by Dual-energy X-ray Absorptiometry scanning (iDXA, Lunar Corporation, Madison, Wisconsin, USA) according to standard procedures. The regions of interests were determined by the encore software (encore software version 14.10.022, GE Medical Systems, Madison, United States). In addition, body composition was evaluated by the whole-body scan: body weight (BW, kg), BMI (kg/m ^2^), body fat percentage (%BF, %), total fat mass (FM, g), total lean body mass (LBM, g), visceral adipose tissue (VAT) mass (g) and volume (m ^3^), and total bone mineral content (BMC, g). The participants were asked to remove metal objects and empty their bladder prior to scanning.

### Blood sampling & biochemical analyses

The plasma concentration of BTM at BASE, after 3wk, and after 19wk of training were measured. After an overnight fast and without any vigorous activities in the previous 48 hours, participants showed up in the laboratory in the early morning. Blood samples were collected from the antecubital vein with a butterfly needle, then transferred to EDTA tubes and centrifuged immediately. The plasma fractions were put on dry ice. Eighteen ml of blood were taken from each participant per test day. Following each test, a sample was placed at -80°C for future P1NP, OC, and CTX analysis, which were assessed by the Chemiluminescence method using a fully automated immunoassay system (iSYS, Immunodiagnostic Systems Ltd., Bolton, England). The assay performance expressed as inter-run variation coefficients were 8% for P1NP, 9% for OC, and 10% for CTX.

### Training status

To estimate the participants’ general training status, a progressive test of maximal oxygen uptake (VO _2_-max) (ml/kg/min) was performed on an electronic bicycle ergometer (Monark 839E, Monark Exercise AB, Vansbro, Sweden) according to standard procedures. A breath-by-breath gas online analyzing system (Jaeger Oxycon Pro, VIASYS Healthcare, Höchberg, Germany) was connected to the participant, and a direct VO _2_-max measurement was conducted.

### Dynamic balance

The “four square step test” (FSST) was performed to evaluate the dynamic balance ^18^ in MMT. The test requires a person to move systematically forward, sideward, backward, and sideward again over four narrow sticks circa 2.5 cm in diameter and 100 cm in length placed on the floor like a cross. The participants were requested to complete the sequence as fast as possible without touching the sticks. Facing the cross, the participants started in the lower left quadrant by stepping forward over the stick into the next left quadrant, stepped sideward over the stick again into the upper right quadrant, and stepped backward over the stick into the lower right quadrant, then stepped sideward over the stick into the left quadrant where they started. Thus, they were moving in a clockwise direction. The floor in each square must be contacted by both feet, and the participants were asked to face forward for the whole sequence. However, the participants were allowed to turn to step into the next square if needed. They started and finished in the same square, and the score was given in seconds (sec). The test was done twice, and the fastest time was used as the FSST score.

### Functional muscle strength and power

The functional muscle strength and power were evaluated in MMT by a jump-and-reach test (vertical jump height) (Vertec Sports Imports, Hilliard, OH). First, the participants were taught how to perform a countermovement jump and how to displace the vanes that was placed above reaching height on a vertical stand. After thoroughly instruction and familiarization, the jump-and-reach test was performed. The maximum vertical jump height (cm) was determined by the difference between the participant standing reach height and the highest displaced vane. The greatest value out of three trials was taken as the result.

### Statistical analysis

All of the statistical analyses were conducted using the Statistical Package for the Social Sciences (SPSS, version 26.0, IBM Corp., Armonk, NY, USA). The standard descriptive statistics and unpaired T-test were used to describe and test the baseline characteristics of the participants. Repeated measurements ANOVA and general linear model with post hoc tests were used to test the effect of training on BTM, BMD, dynamic balance, and the jump-and-reach test. A p-value < 0.05 was considered significant. Unless otherwise stated, values are given as mean ± standard error (SE).

## Results

### Participant characteristics

At baseline, there were significant differences between MMT and CON for BW, BMI, FM, left and mean femur BMD values, OC, and CTX ( Table 1).

**Table 1.** Baseline characteristics of the participants.

	MMT (n = 9)	CON (n = 13)	p-value
Age (years)	62.4 ± 3.8	58.7 ± 4.7	0.061
Height (cm)	166.9 ± 6.4	166.9 ± 5.2	0.989
Weight (kg)	72.8 ± 5.6	64.2 ± 10	0.022 *
BMI (kg/m ^2^)	26.3 ± 2.2	22.9 ± 2.9	0.009 *
Body Fat Percentage (%)	39.4 ± 3.1	33.7 ± 8.5	0.070
Total Fat Mass (kg)	28.7 ± 2.7	22.2 ± 8.6	0.043 *
Total Lean Body Mass (kg)	41.8 ± 4.3	39.8 ± 3.4	0.233
VAT-mass (g)	793 ± 351.2	511 ± 434.8	0.123
VAT-volume (cm ^3^)	840.6 ± 372.2	541.7 ± 460.9	0.123
Total BMC (g)	2266.9 ± 206.5	2067.2 ± 300.2	0.100
BMD (g/cm ^2^)			
Whole-body	1.121 ± 0.073	1.046 ± 0.099	0.066
Lumbar spine	1.088 ± 0.137	1.014 ± 0.111	0.175
Right Femur neck	0.918 ± 0.092	0.834 ± 0.097	0.057
Left Femur neck	0.941 ± 0.088	0.823 ± 0.076	0.003 *
Mean Femur neck	0.929 ± 0.089	0.829 ± 0.085	0.015 *
Right Total Femur	0.947 ± 0.098	0.864 ± 0.091	0.056
Left Total Femur	0.949 ± 0.101	0.840 ± 0.091	0.016 *
Mean Total Femur	0.948 ± 0.098	0.852 ± 0.090	0.028 *
Right Trochanter	0.741 ± 0.087	0.683 ± 0.092	0.149
Left Trochanter	0.745 ± 0.091	0.661 ± 0.097	0.055
Mean Trochanter	0.743 ± 0.086	0.672 ± 0.093	0.084
Right Shaft	1.151 ± 0.126	1.046 ± 0.125	0.068
Left Shaft	1.149 ± 0.131	1.008 ± 0.121	0.017 *
Mean Shaft	1.150 ± 0.126	1.027 ± 0.121	0.033 *
BTM			
P1NP (μg/l)	52.8 ± 16.6	62.1 ± 21.3	0.298
OC (μg/l)	19 ± 6.5	28.9 ± 7.1	0.003 *
CTX (ng/l)	331.2 ± 100.7	627.8 ± 253.8	0.001 *
VO _2_-max (ml/min/kg)	28.2 ± 3.6	30.5 ± 4.7	0.204

Values are given as means ± SD. BMI = body mass index, BMD = bone mineral density, BTM = bone turnover marker.

* Significant difference between groups (p < 0.05). Unpaired T-test.

### Attendance and dropout

As described in the participants section, two persons in MMT dropped out due to personal reasons, and four participants did not meet the mean attendance criterion of at least 1 session weekly (>19 sessions) and were subsequently excluded in the data analyses. The mean attendance rate for the remaining participants (9 women) was 24.2 sessions (65.5%) of the maximum number of sessions offered (37 sessions). The individual attendance rates were 20-30 sessions (54.1-81.1%). with a decrease in attendance rate over time from 6.2 out of 8 sessions (78%) in the first four-week period and 5.1 out of 8 sessions (64%) in the last four-week period.

The attendance rate range (54.1-81.1%) in the present study showed that there is inter-individual variability. The participants attended the training more in the first than the last four-week.

### BMD and body composition

**Figure 2.**
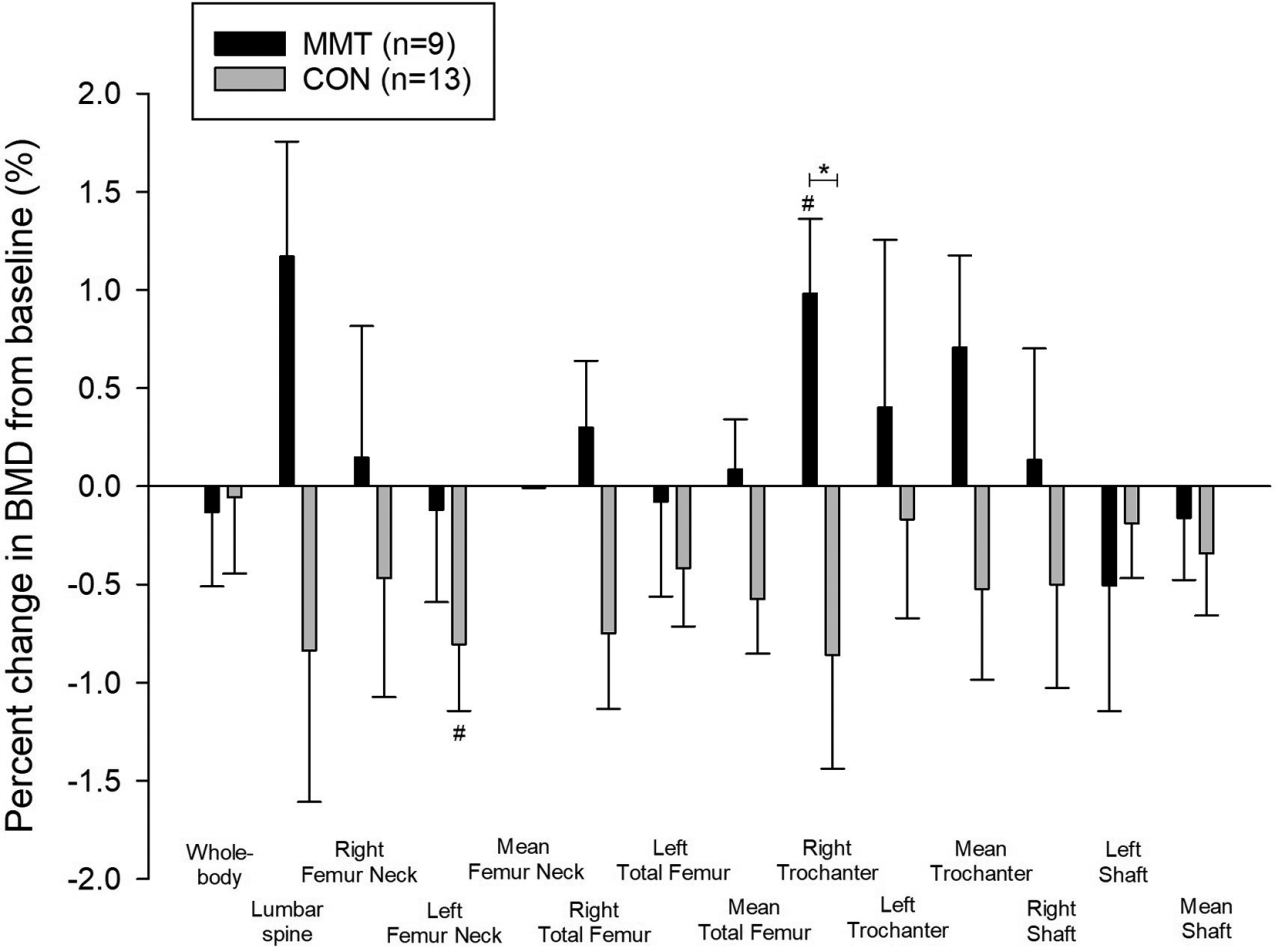
Percent change (± SE) in whole-body and regional bone mineral density (BMD) following the 19-week multimodal training. *Significant between-group difference (p < 0.05). #Significant within-group difference (p < 0.05).

After 19wk of training, right trochanter BMD (g/cm ^2^) increased significantly from baseline for MMT (1.0%, p = 0.03), which was significantly different from CON (p = 0.03) ( Table 2), but there was no significant BMD change in any other region. However, LS (1.2%) and right total femur (0.3%) tended to increase (both, p < 0.1) for MMT ( Figure 2). Whole-body BMC decreased significantly (0.7%, p = 0.016) within CON.

**Table 2.** Absolute BMD values (Mean ± SD).

	MMT (n = 9)		CON (n = 13)		p-value ^a^
	Baseline	19wk	Baseline	19wk	
Whole body	1.121 ± 0.073	1.119 ± 0.070	1.046 ± 0.099	1.045 ± 0.099	0.873
Lumbar spine	1.088 ± 0.137	1.100 ± 0.128	1.014 ± 0.111	1.005 ± 0.110	0.067
Right Femur neck	0.918 ± 0.092	0.920 ± 0.104	0.834 ± 0.097	0.829 ± 0.088	0.359
Left Femur neck	0.941 ± 0.088	0.939 ± 0.083	0.823 ± 0.076	0.817 ± 0.079 ^#^	0.337
Mean Femur neck	0.929 ± 0.089	0.929 ± 0.092	0.829 ± 0.085	0.823 ± 0.081	0.149
Right Total Femur	0.947 ± 0.098	0.950 ± 0.104	0.864 ± 0.091	0.857 ± 0.086	0.054
Left Total Femur	0.949 ± 0.101	0.947 ± 0.088	0.840 ± 0.091	0.837 ± 0.092	0.763
Mean Total Femur	0.948 ± 0.098	0.948 ± 0.094	0.852 ± 0.090	0.847 ± 0.088	0.136
Right Trochanter	0.741 ± 0.087	0.749 ± 0.090 ^#^	0.683 ± 0.092	0.677 ± 0.093	0.026 *
Left Trochanter	0.745 ± 0.091	0.746 ± 0.073	0.661 ± 0.097	0.660 ± 0.099	0.771
Mean Trochanter	0.743 ± 0.086	0.747 ± 0.077	0.672 ± 0.093	0.669 ± 0.095	0.129
Right Shaft	1.151 ± 0.126	1.154 ± 0.140	1.046 ± 0.125	1.040 ± 0.118	0.333
Left Shaft	1.149 ± 0.131	1.142 ± 0.117	1.008 ± 0.121	1.006 ± 0.121	0.502
Mean Shaft	1.150 ± 0.126	1.148 ± 0.126	1.027 ± 0.121	1.023 ± 0.118	0.681

Notes: BMD values indicate g/cm ^2^. MMT = Multimodal training group, CON = Control group.

^a^ General Linear Model + post hoc test.

* Significant difference between group (p < 0.05).

# Significant difference within group (p < 0.05).

For body composition, there were significant reductions in %BF (4.0%, p = 0.006), FM (6.0%, p = 0.013), VAT-mass (11.3%, p = 0.004), VAT-volume (11.3%, p = 0.004) within the MMT compared to BASE ( Table 3). Compared to CON, there were significant differences for %BF (p = 0.049), FM (p = 0.021), VAT-mass (p = 0.013), VAT-volume (p = 0.013), and whole-body BMC (p = 0.041) delta values.

**Table 3.** Body composition values (Mean ± SD).

	MMT (9)		CON (n = 13)		p-value ^b^
	Baseline	19wk	Baseline	19wk	
Weight ^a^ (kg)	72.8 ± 5.6	71.2 ± 5.3	64.2 ± 10.3	63.8 ± 10.3	0.162
BMI (kg/m ^2^)	26.3 ± 2.2	25.9 ± 2.4	22.9 ± 2.9	22.9 ± 3	0.258
%BF (%)	39.4 ± 3.1	37.8 ± 2.7 #	33.7 ± 8.5	33.5 ± 8	0.049 *
Total Fat Mass (kg)	28.7 ± 2.7	26.9 ± 2.7 #	22.2 ± 8.6	22 ± 8.3	0.021 *
Total Lean Mass (kg)	41.8 ± 4.3	42 ± 3.8	39.8 ± 3.4	39.7 ± 3.4	0.673
VAT-mass (g)	793 ± 351.2	694 ± 307 #	511 ± 434.8	508.8 ± 421.5	0.013 *
VAT-volume (cm ^3^)	840.6 ± 372.2	735.7 ± 325.4 #	541.7 ± 460.9	539.4 ± 446.8	0.013 *
Whole-body BMC (g)	2266.9 ± 206.5	2269.8 ± 202.4	2067.2 ± 300.2	2052.6 ± 301.3 #	0.041 *

MMT = Multimodal training group, CON = Control group, BMI = Body Mass Index.

^a^ Geometric mean.

^b^ General Linear Model + post hoc test.

* Significant difference between groups (p < 0.05).

# Significant difference within-group (p < 0.05).

### BTM

**Figure 3.**
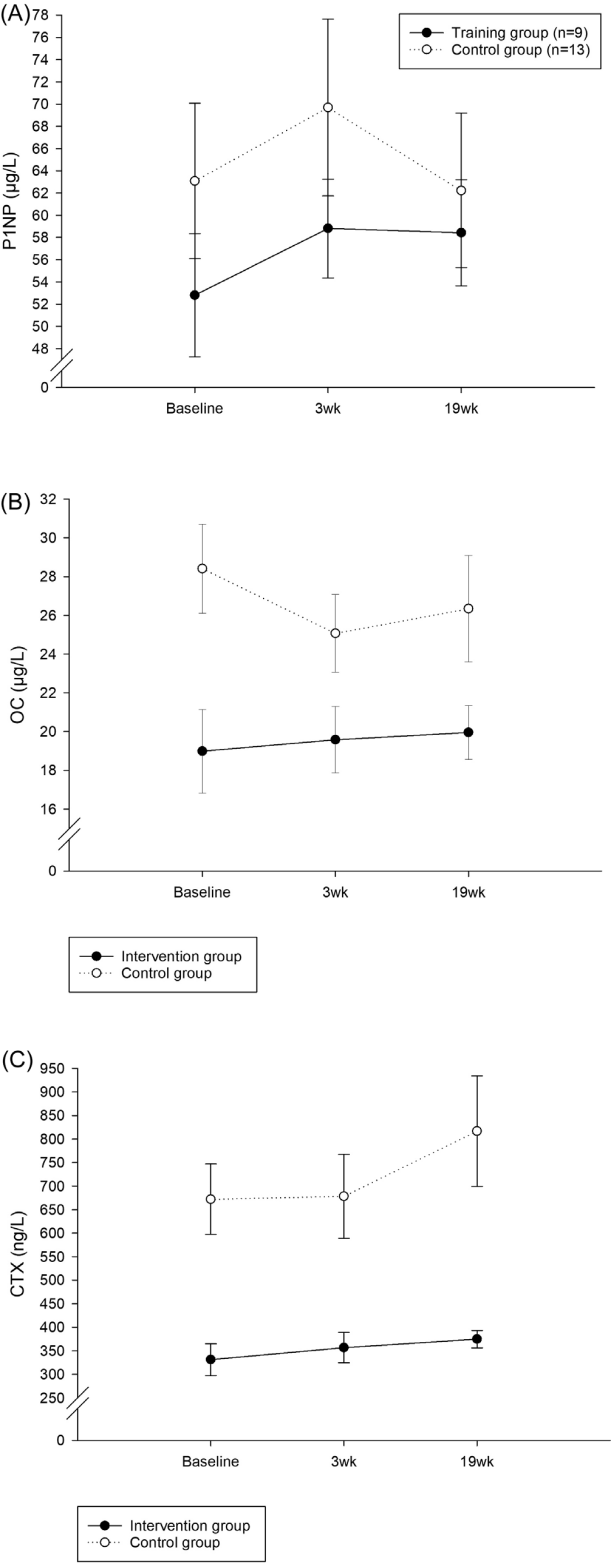
Bone turnover markers. (A) Procollagen type-1 amino-terminal propeptide (P1NP), (B) osteocalcin (OC), and (C) C-terminal telopeptide of type-1 collagen (CTX). Time measurements: Immediately before exercise (baseline), after 3 weeks (3wk), after 19 weeks (19wk). The lines represent means ± SE.

The plasma concentrations of P1NP, OC, and CTX are shown in Figure 3 and Table 4. There was no significant within- or between-group difference observed at 3wk or 19wk compared to baseline for all markers.

**Table 4.** Bone turnover marker (BTM) concentrations at BASELINE, after 3 weeks of training (3wk), and after 19 weeks of training (19wk).

Outcome	Group	BASELINE	3wk	19wk	p-value within-group	p-value between-group
**Markers of bone formation**						
**P1NP (μg/L)**	MMT (n = 9)	52.8 ± 5.5	58.8 ± 4.5	58.4 ± 4.8	0.193	0.304
	CON (n = 13)	62.1 ± 5.9	69.7 ± 7.3	62.4 ± 5.9	0.218	
**OC (μg/L)**	MMT (n = 9)	19.0 ± 2.2	19.6 ± 1.7	20.0 ± 1.4	0.810	0.231
	CON (n = 13)	28.9 ± 2.0	25.1 ± 1.8	24.8 ± 2.6	0.173	
**Marker of bone resorption**						
**CTX (ng/L)**	MMT (n = 9)	331.2 ± 33.6	356.6 ± 32.2	374.8 ± 18.5	0.330	0.394
	CON (n = 13)	627.8 ± 70.4	678.2 ± 82.1	729.5 ± 115.8	0.243	

Notes: All values are expressed as mean ± SE. P1NP = procollagen type-1 amino-terminal propeptide, OC = Osteocalcin, CTX = C-terminal telopeptide of type 1 collagen, MMT: Multimodal training, CON: Control. Repeated measurement ANOVA.

### FSST & Jump-and-reach test

There were no significant changes in both FSST and jump-and-reach test at 19-week compared to baseline for MMT ( Table 5). However, there was a tendency to be faster in FSST at 19-week (p = 0.06).

**Table 5.** Dynamic balance and functional muscle strength result for multimodal training group (n=9). Paired t-test.

	Mean ± SD	Difference ± SD	CI-95%	p-value
Four Squared Step Test (sec)				
Baseline	7.5 ± 1.0	0.6 ± 0.8	−0.04 – 1.2	0.062
19wk	6.9 ± 0.8			
Jump and reach test (cm)				
Baseline	20.2 ± 6.1	−1.3 ± 3.7	−4.2 – 1.5	0.308
19wk	21.6 ± 6.3			

## Discussion

The present study aimed at evaluating the feasibility and whether 19wk of multimodal training offered as musculoskeletal health promotion in a local community had an effect on musculoskeletal health, body composition, dynamic balance, and functional muscle strength in postmenopausal women. The main finding from this study was that the training program had a positive effect on body composition. Thus, %BF, FM, VAT-mass, and VAT-volume were significantly reduced in MMT compared to CON, showing that the body composition change was due to the training program. In addition, the right trochanter BMD was improved for MMT, and the whole-body BMC was maintained for MMT in contrast to a significant reduction in CON.

This study found that only right trochanter BMD was significantly increased (1.0%, p < 0.05) in MMT. There was no significant changes in other regions of BMD. This finding could be due to the participant attendance did not meet the recommendations of training intensity, volume, and frequency. According to Daly and Giangregorio, ^19^ the minimum frequency of resistance training is at least two days per week and for weight-bearing impact exercise 4-7 times per week, which the present study not met as no participant fulfilled 37 times in total for 19wk of training.

The whole-body BMC was not changed in MMT, whereas it was significantly decreased (p = 0.02) by 0.7% in CON, which was significantly (p = 0.04) different from MMT ( Table 3). Thus, it seems from the present study that the training at “ *Knoglestærk*” maintained total bone mass, even with a required minimum training attendance of just one hour weekly. The loss of whole-body BMC (−0.7%, p = 0.016) in CON group is consistent with Gallagher *et al*., ^20^ who investigated the effect of age and menopause status on bone. The finding that the change in BMC differed between MMT and CON is not in line with Mosti *et al*., ^16^ who investigated the impact of three sessions of squat exercise maximal strength training in postmenopausal women with osteoporosis or osteopenia (mean age: 61.9 ± 5.0 y) for 12 weeks. Moreover, they found an increase in lumbar spine BMC not whole-body. Thus, they found an increase of 2.9 ± 2.8% (p = 0.01) LS BMC from baseline in training group, and this change was higher (p = 0.03) than in control group.

The reduction in %BF and FM ( Table 3) in this study is in agreement with Daly *et al*., ^21^ who investigated the effect of one year supervised and structured multi-component training program in community-dwelling independently living men (27%) and women (73%) (mean age: 67.4 y, 60-86 years). They found that the FM was significantly decreased (p < 0.001) by 3.2%, which is lower than the present study (6.0%, p = 0.01). The higher reduction in the present study compared to Daly *et al*. ^21^ study might be due to the younger participant (62.4 ± 3.8 y) which has a higher metabolic rate than the older one (66 – 94 y), ^22^ that could influence the change of body fat. The reduction in %BF and FM is in line with Nielsen *et al*. ^23^ as well, who investigated the feasibility and physiological health effects of 15-week training performed minimum once a week for sedentary elderly. They found that the total FM was significantly decreased (−2.0 kg, p = 0.01) and the %BF as well (-1.6%, p = 0.01).

As the VAT assessment by iDXA is a fairly new functionality, only very few studies report results for this outcome, and it seems that none has studied training effects on VAT for healthy postmenopausal women. Moreover, no normal reference intervals for specific populations are established yet. The present finding of a 11.3% reduction in VAT seems in line with the Yen *et al*. study, ^24^ which reported a 6.3% decrease in VAT after eight weeks of aerobic and resistance exercise intervention. However, the study participants were a group of male and female patients receiving chemotherapy for head and neck cancer (mean age: 52 ± 10.7 y). As reported by Yen *et al*. ^24^ the lower response than the present study (-6.3% vs. −11.3%) may be due to the disease or the shorter duration (8wk vs. 19wk), even though their intervention more frequent (3 days/week vs. one day/week). ^25^ Moreover, it could be hypothesized that the participants in Yen *et al*. ^24^ study had a lower %BF and VAT at baseline, therefore, it is more difficult to induce a training response. Nevertheless, more studies still needed on healthy postmenopausal women.

Despite the favorable result, the present study has several limitations. The dietary intakes were not recorded. Thus, the changes that occur in the body composition could not be known whether entirely due to the training or dietary intake of participants. Future studies should consider taking the dietary intake history and monitoring habitual daily physical activity, which may add an explanation regarding the changes specifically in body composition in the present study. Another thing to note is that the *Knoglestærk* health promotion program is a real-life setting and thus the training is not as well controlled as in a laboratory setting. Because neither heart rate (HR) nor accelerometer measurements were recorded during exercise, it cannot be ruled out that the individual training intensity varied among the participants. Hence, the use of HR monitor and accelerometer would improve the control and the standardization of the training and thus reduce the variation in the individual response and the reliability of the results. In addition, we did not carry out a priori power analyses. The power calculations were performed after the studies had been conducted (provided as *Extended data* ^26^), and the statistical power of the present study seems to be underpowered. Therefore, a larger sample size would have a positive effect on the effect sizes due to a reduction in standard error and thus an improvement of the statistical power. The effect size of the intervention was varied, with greatest effect on VAT-volume & masse, total BMC, weight, total fat mass and body fat percentage. On the contrary, the intervention has smaller effect on general and specified BMDs. The given amount of time (19wk) and specified intervention (multimodal training group) had greater effect on fat changes rather than bone changes. The interesting part was the total BMC that affected by the intervention was not followed by BMD's changing.

## Conclusions

The results of the present study indicate that while 19 weeks of multimodal musculoskeletal training as offered by the *Knoglestærk* health promotion training initiative is feasible for postmenopausal women by inducing health-enhancing effects on body composition, however due to the attendance compliance, the training did only elicit minor significant improvements in BMD, thus an increase was found in right trochanter BMD and whole-body BMC. No changes in dynamic balance or the functional muscle strength were observed in the present study. To promote musculoskeletal improvements for all participants, a better control of the individual musculoskeletal training intensity, frequency, and attendance rate is highly recommended.

## Data availability

### Underlying data

Figshare: Underlying data for ‘A Feasibility Study of Training in a Local Community Aimed Upon Health Promotion with Special Emphasis on Musculoskeletal Health Effects.’

https://doi.org/10.6084/m9.figshare.16836940. ^29^

This project contains the following underlying data:

- A Feasibility Study of Training Dataset1.csv (dataset of participant characteristic, body composition, and bone mineral density)
- A Feasibility Study of Training Dataset2.csv (dataset of participants’ bone turnover markers)
- A Feasibility Study of Training Dataset3.csv (dataset of functional muscle strength and power and dynamic balance result)

### Extended data

Figshare: Working title: A Feasibility Study of Training in a Local Community Aimed Upon Health Promotion with Special Emphasis on Musculoskeletal Health Effects. Extended data - Statistical power of the paired t-tests for body composition, regional BMD, and bone turnover markers. https://doi.org/10.6084/m9.figshare.16611655.v1 ^26^

Figshare: Working title: A Feasibility Study of Training in a Local Community Aimed Upon Health Promotion with Special Emphasis on Musculoskeletal Health Effects. Extended data - Effect size of the Training Program. https://doi.org/10.6084/m9.figshare.16611787.v1 ^27^

### Reporting guidelines

Figshare: CONSORT checklist, extension for Pilot and Feasibility Trials for study “A Feasibility Study of Training in a Local Community Aimed Upon Health Promotion with Special Emphasis on Musculoskeletal Health Effects”. https://doi.org/10.6084/m9.figshare.16610341.v1 ^28^

Data are available under the terms of the Creative Commons Zero “No rights reserved” data waiver (CC0 1.0 Public domain dedication).
