# Supplementary material for: Feasibility Study of Community-Based Training for Musculoskeletal Health Promotion
Source: F1000Res. 2024 Sep 4;11:51. Originally published 2022 Jan 17. [Version 3] doi: 10.12688/f1000research.73698.3 (PMC11387933; doi:10.12688/f1000research.73698.3)
Supplement: Peer review history

# Reviewer report: Reviewer response for version 3

**DOI:** 10.5256/f1000research.171086.r320739

**Published:** 2024-09-10

**Authors:** Jan Gajewski

**Affiliations:** 1 Faculty of Physical Education, Jozef Pilsudski University of Physical Education, Warsaw, Poland

I read the paper and I am satisfied with the changes introduced. I thank the authors for their responses and have no further comments.

Is the work clearly and accurately presented and does it cite the current literature?

Yes

If applicable, is the statistical analysis and its interpretation appropriate?

Partly

Are all the source data underlying the results available to ensure full reproducibility?

Yes

Is the study design appropriate and is the work technically sound?

Partly

Are the conclusions drawn adequately supported by the results?

Yes

Are sufficient details of methods and analysis provided to allow replication by others?

Partly

Reviewer Expertise:

Sport science, biomechanics, statistics

I confirm that I have read this submission and believe that I have an appropriate level of expertise to confirm that it is of an acceptable scientific standard.

# Reviewer report: Reviewer response for version 2

**DOI:** 10.5256/f1000research.144978.r279862

**Published:** 2024-05-31

**Authors:** Jan Gajewski

**Affiliations:** 1 Faculty of Physical Education, Jozef Pilsudski University of Physical Education, Warsaw, Poland

The purpose of the study was to evaluate the feasibility of a 19-week multimodal training in a local community center targeting musculoskeletal health in postmenopausal women. The study was designed as a controlled trial. In the end, only 9 subjects in the experimental group and 13 in the control group completed participation in exercise and measurement.

 I like the idea itself very much. Evaluation of the effectiveness of the exercise program in the real conditions of the local community shows the practical usefulness of similar initiatives. The feasibility of the established program turned out to be moderately satisfactory.  The effects of the program have not been fully proven. This had to do primarily with the relatively small size of the groups. I checked with G*Power that the minimum sizes to detect a moderate effect (eta squared>0.06) at 80% statistical power are at least 17 people in each group. When planning the experiment, a correspondingly larger number of subjects should have been taken into account (e.g. 22 and 22). I understand that for various reasons it was not possible. It was unfortunate that the groups differed significantly in the base line parameters (especially in age, bone turnover markers. Of course it cannot be changed.

 I have reservations about the statistical methods used or rather about presentation and interpretation of their outcomes. For an experiment, the most important result is the interaction of a fixed factor (Group) and a repeated factor (please report F, p and eta squared). If this interaction occurs, one can talk about a different response in the groups. This should not necessarily focus on the results of post-hoc tests. However, the authors focused on within-group and between-group differences which is not as important as interaction.

 As I had some time I recalculated the results again and attached them in an excel file please refer to this link: *https://f1000research.s3.amazonaws.com/linked/651424.A_Feasibility_Study_of_Training_Dataset_ver_1_1_.xlsx*

 When the variables involved left and right, I expanded the analysis to include a second factor repeated SIDE. I didn't check the normality of the distributions, but it should probably be done. It would be worthwhile to devote some space to the effect sizes (eta squared). In some cases, the interactions were close to significance with relatively large effect sizes. Unfortunately, due to low statistical power (group sizes), such results have low reliability but at least there is something to discuss.

 I would also ask the authors to expand on the description of the exercises used. Based on the description, it would not be possible to reproduce the intervention exactly.

 Specific comments

 In Table 2, the p-value evaluates the group effect, which is not valid in this case. I would rather put F, p and eta2 for the interaction in the last column. Asterisks could be used to indicate any differences between groups in individual measurements (different than control).

 The same goes for Table 3. Why does the designation ‘a-geometric mean’ at body weight appear (what's the reason)?

 Body composition parameters are shown twice in Table 1 and Table 3.

 Aren't the results shown in Figure 3 duplicated in Table 4? Let's stick to the rule that each result is presented once in one of three ways: in text, in a table or on a graph.

 Summing up

 Of course, the article has irremovable flaws related to the allocation into groups and their size, but if the practical aspect of the results obtained is taken into account, the value of the study increases.

Is the work clearly and accurately presented and does it cite the current literature?

Yes

If applicable, is the statistical analysis and its interpretation appropriate?

Partly

Are all the source data underlying the results available to ensure full reproducibility?

Yes

Is the study design appropriate and is the work technically sound?

Partly

Are the conclusions drawn adequately supported by the results?

Yes

Are sufficient details of methods and analysis provided to allow replication by others?

Partly

Reviewer Expertise:

Sport science, biomechanics, statistics

I confirm that I have read this submission and believe that I have an appropriate level of expertise to confirm that it is of an acceptable scientific standard, however I have significant reservations, as outlined above.

# Response

**Published:** 2024-08-27

**Authors:** Rizky Suganda Prawiradilaga

Dear Reviewer,

 Thank you for your thorough and insightful review of our manuscript. We appreciate the time and effort you have taken to evaluate our work and provide valuable feedback.

 We would like to address some of the key points you raised:

**1. Statistical Methods:**

 •    We chose to use GLM to evaluate the interaction between the treatment (Multimodal Training, MMT) and time of observation, focusing particularly on musculoskeletal health outcomes. We recognize the limitations posed by our small sample size, yet the GLM analysis revealed a significant interaction between MMT and time observed in increasing bone density in the right trochanter. This finding underpinned our conclusion that the treatment was effective in this area.

 •    We did not employ repeated measures ANOVA because the assumptions necessary for this analysis were not met by our data. GLM allowed us to account for both fixed and repeated factors, making it a more appropriate choice given the design and characteristics of our study. However, we appreciate your recommendation to emphasize the interaction effects and will adjust our manuscript accordingly.

**2. Presentation of Results:**

 •   In line with your suggestion, we have revised the relevant tables to include F-statistics, p-values, and eta-squared values for the interaction effects between group and time. These revisions will help clarify the significance of these interactions and better align with standard reporting practices.

 •    We acknowledge the concern about the low statistical power due to the small sample size, which indeed limits the reliability of some findings. We will include a more detailed discussion of effect sizes (eta squared) and their implications, as well as the potential limitations related to power in our revised manuscript.

**3. Description of the Exercises:**

 •    Following your feedback and based on detailed information from our related research, we have expanded the description of the exercises used in the study. The training program included high- and odd-impact exercises, progressive resistance training, and balance and coordination activities. We have now provided a comprehensive explanation of these activities, including their intensity, repetitions, and the rationale behind their inclusion, ensuring that the intervention can be reproduced in future studies.

**4. Data Presentation:**

 •    We have revised the tables and figures to ensure clarity and avoid unnecessary duplication. Specifically, we have deleted Figure 3 to prevent the repetition of results already presented in Table 4. Regarding the body composition parameters, we recognize that Tables 1 and 3 serve different purposes. Table 1 provides a summary of the baseline characteristics of the study participants, including body composition metrics, to offer context for the starting points of each group. Table 3, on the other hand, presents the changes in body composition parameters throughout the intervention, highlighting the outcomes of the study. Therefore, while there may be some overlap in the parameters listed, each table is designed to address distinct aspects of the study, and the information provided in both tables is essential for a comprehensive understanding of the results.

**5. The designation ‘a-geometric mean’ at body weight (what's the reason?)**

 •   In the context of our study, body weight data may have been log-transformed to normalize the distribution before analysis, and the geometric mean was used to summarize these values appropriately. The ‘a-geometric mean’ designation was included to clarify that the mean values reported are not simple arithmetic means but are geometric means, which are more appropriate for data with a skewed distribution.

**6. Summary and Conclusion:**

 •    While we recognize that the small sample size and differences in baseline characteristics between groups represent inherent limitations in our study, we believe that the practical implications of our findings contribute valuable insights to community-based health promotion programs. We are committed to enhancing the clarity and rigor of our manuscript in light of your recommendations.

 Thank you once again for your constructive feedback. We believe these revisions will significantly improve the quality of our manuscript, and we appreciate your continued consideration.

 Best regards,

 Rizky Suganda Prawiradilaga

# Reviewer report: Reviewer response for version 2

**DOI:** 10.5256/f1000research.144978.r165893

**Published:** 2023-03-22

**Authors:** Robert Wen-Wei Hsu

**Affiliations:** 1 Department of Orthopaedic Surgery, Chang Gung Memorial Hospital, Pu-Tz City, Taiwan

No further comments to make.

Is the work clearly and accurately presented and does it cite the current literature?

No

If applicable, is the statistical analysis and its interpretation appropriate?

Yes

Are all the source data underlying the results available to ensure full reproducibility?

No

Is the study design appropriate and is the work technically sound?

No

Are the conclusions drawn adequately supported by the results?

Partly

Are sufficient details of methods and analysis provided to allow replication by others?

No

Reviewer Expertise:

Orthopedics, sports medicine

I confirm that I have read this submission and believe that I have an appropriate level of expertise to confirm that it is of an acceptable scientific standard.

# Response

**Published:** 2023-04-22

**Authors:** Rizky Suganda Prawiradilaga

Dear Reviewer,

 We would like to say thank you for your valuable comments and suggestions that have improved the manuscript considerably.

 Best Regards

# Reviewer report: Reviewer response for version 1

**DOI:** 10.5256/f1000research.77367.r152766

**Published:** 2022-11-08

**Authors:** Robert Wen-Wei Hsu

**Affiliations:** 1 Department of Orthopaedic Surgery, Chang Gung Memorial Hospital, Pu-Tz City, Taiwan

Thank you for sending this paper for reviewing. There were some comments on the following:

1. The sample size is too small to validate the effect of training.
2. How was the sample size determined?
3. Statistical power should be calculated.
4. The exercise program is not strong enough to have physiological impact to the health.
5. The duration of exercises is not long enough.
6. The study is required to do prospective study. The assignment to exercise and control group should be randomized.

Is the work clearly and accurately presented and does it cite the current literature?

No

If applicable, is the statistical analysis and its interpretation appropriate?

Yes

Are all the source data underlying the results available to ensure full reproducibility?

No

Is the study design appropriate and is the work technically sound?

No

Are the conclusions drawn adequately supported by the results?

Partly

Are sufficient details of methods and analysis provided to allow replication by others?

No

Reviewer Expertise:

Orthopedics, sports medicine

I confirm that I have read this submission and believe that I have an appropriate level of expertise to state that I do not consider it to be of an acceptable scientific standard, for reasons outlined above.

# Response

**Published:** 2023-02-28

**Authors:** Rizky Suganda Prawiradilaga

1. We are aware of that. We tried to equate the number of samples with our previous studies but, in our feasibility study with community settings, it turned out that 6 people from the MMT group dropped out.
2. The sample size was determined by the Author judgement based on the previous study on Bone Turnover Markers ( Prawiradilag *et al. *(2020)).
3. The Statistical power calculations were performed with Power and Sample Size Calculations version 3.0 for the paired t-test and performed with SPSS for the repeated ANOVA measurement. The results are in the link in the ‘Extended data’ section.
4. We apologize; we do not actually understand, what do you mean by not strong enough? The Health initiative program uses multimodal training including 1) High- and odd-impact exercise including multi-directional games; 2) Progressive resistance training (PRT); 3) Balance and coordination training which has an osteogenic impact based on evidence, but we are aware that frequency, duration did not meet the recommendation. In addition, we didn’t measure the heart rate for intensity.
5. We understand that the Health Promotion initiative program was not long enough (19 weeks) and did not meet the recommendation (6-12 months) for osteogenic exercise. We would like to see whether there were bone turnover markers (BTM) changes in this short period since, in our previous study, there were acute responses in BTM.

Thank you for your suggestions.

- Is the work clearly and accurately presented and does it cite the current literature?

We have cited the current literature

- Is the study design appropriate and is the work technically sound?

We have added, “This is a feasibility study in a local community with a non-randomized controlled trial approach.”

- Are sufficient details of methods and analysis provided to allow replication by others?

We think it is sufficient since the study method and the training program were described clearly.

- Are all the source data underlying the results available to ensure full reproducibility?

We share the raw data in the ‘Extended data’ section.

# Reviewer report: Reviewer response for version 1

**DOI:** 10.5256/f1000research.77367.r134828

**Published:** 2022-05-16

**Authors:** Melanie Kistler-Fischbacher

**Affiliations:** 1 Menzies Health Institute Queensland, Griffith University, Gold Coast, Qld, Australia

This non-randomised controlled study reports the effect of a 19-week multicomponent exercise intervention (resistance, balance and impact training) on bone markers, bone mineral density and body composition outcomes among 28 postmenopausal women. I have concerns about the study design and methodological rigor of the trial. Specifically, the duration of the trial 19 weeks is insufficient to detect changes in bone, bias associated with the differences between groups at baseline and the lack of adjustment for multiple comparisons.

**Introduction**

 Paragraph (P) 1: “is it estimated that 200 million females…”. This number actually includes males and females.

 P2: “One way to prevent… age-related bone loss”. This is a very old reference. Since 1987 a large number of exercise trials for bone have been conducted. I suggest including a more recent reference.

 P3 & 4: In para 3 you hypothesize that “evidence-based multimodal training will have an osteogenic effect and in para 4 you state that there is a need for “evidence-based trainings”. This is contradicting and I don’t think you can call this MMT “evidence based”

 P4: “Only few studies have examined…”. There has been a large number of studies that have tested combined impact, resistance and balance training interventions in recent years which showed safety and efficacy. ^^1^, ^2^, ^3^, ^4^^ These should be cited here plus the existing gap in the literature identified.

 P5: “The aim was to investigate the feasibility…” Feasibility is mentioned in the title, the aims and discussion (e.g. discussion 1 ^st^ sentence and conclusion 1 ^st^ sentence), however, it is unclear how feasibility was measured. Feasibility is also not listed as an endpoint (e.g. clinicaltrials.gov). Please clarify.

**Methods**

 General: please clarify which outcomes were primary and which ones were secondary.

 P1: Please explain how participants were recruited, particularly the sedentary control group.

 P1: The duration of 19 weeks is inappropriate to measure changes in bone mineral density. A minimum of 6-8 months is needed to detect changes in bone.

 P3: “to ensure transparency” The trial was registered close to manuscript submission data, which does not ensure transparency. I suggest deleting this part of the sentence.

 P4: How was sedentary defined?

- Was there a minimum age set?
- How was postmenopausal defined? (e.g. how many years post menopause?)
- How was regular, systematic training defined?

P5: The information on the number of participants and drop outs should be moved to the results section.

 P5: “…12 from a previous study...”. Please describe the control group somewhere.

 P8: How many sets and repetitions were performed of each exercise, and how did you ensure the resistance training was progressive?

 P9: Can you include information on the short-term measurement variability of BMD and body composition measures in your lab?

 P12/13: Why were FSST and jump-and-reach tests not performed in the control group?

 P14: Given the large number of outcomes, adjustment for multiple comparison is strongly recommended.

**Results**

 P1: There are significant baseline differences between groups which is of great concern. How did you address this, were analyses adjusted for baseline values?

 P4: “… LS and total femur… tended to increase”. Reporting of trends is controversial; I suggest removing this sentence.

 Table 2: Please explain why you are reporting mean femur BMD in addition to right and left femur values.

 Table 2/3: Your findings show a decrease in whole body BMC and an increase in BMD. How would you explain these differences.

**Discussion**

 Rather than comparing present findings to individual studies, I would recommend reviewing the large body of systematic reviews and meta-analyses on the topic. Many of those have examined the influence of training parameters (e.g. intensity, volume and frequency. ^^3^, ^5^^) Interpretation of your findings in context of those findings would be helpful. For example, recent studies have shown that exercise intensity is positively associated with bone changes, and that low-intensity exercise is ineffective.

 There are a number of limitations of the present study that have not been addressed in the discussion. E.g. bias related to baseline differences, not randomised, only short term (< 6 months), generalisability.

 You state that no power calculation was undertaken. 1) How was the sample size determined? 2) How was a posteriori power calculated and what were the results?

 I would like to see a section about clinical significance of the findings.

**Conclusion**

 “… inducing health-enhancing effects”. In light of the significant baseline differences, other study limitations and the fact that only body composition variables (which were significantly different between groups at BL) improved, I think this is overstating the findings of the study.

Is the work clearly and accurately presented and does it cite the current literature?

No

If applicable, is the statistical analysis and its interpretation appropriate?

Partly

Are all the source data underlying the results available to ensure full reproducibility?

Yes

Is the study design appropriate and is the work technically sound?

No

Are the conclusions drawn adequately supported by the results?

Partly

Are sufficient details of methods and analysis provided to allow replication by others?

No

Reviewer Expertise:

Exercise science; bone; bone-specific exercise; osteoporosis; weight lifting for bone and muscle health; postmenopausal women

I confirm that I have read this submission and believe that I have an appropriate level of expertise to state that I do not consider it to be of an acceptable scientific standard, for reasons outlined above.

## References

[1] : High-Intensity Resistance and Impact Training Improves Bone Mineral Density and Physical Function in Postmenopausal Women With Osteopenia and Osteoporosis: The LIFTMOR Randomized Controlled Trial. *J Bone Miner Res* .33(2) : 10.1002/jbmr.3284 211-220 10.1002/jbmr.3284 28975661

[2] : A Comparison of Bone-Targeted Exercise With and Without Antiresorptive Bone Medication to Reduce Indices of Fracture Risk in Postmenopausal Women With Low Bone Mass: The MEDEX-OP Randomized Controlled Trial. *J Bone Miner Res* .36(9) : 10.1002/jbmr.4334 1680-1693 10.1002/jbmr.4334 34033146

[3] : The effect of exercise intensity on bone in postmenopausal women (part 2): A meta-analysis. *Bone* .143: 10.1016/j.bone.2020.115697 115697 10.1016/j.bone.2020.115697 33357834

[4] : A multi-component exercise regimen to prevent functional decline and bone fragility in home-dwelling elderly women: randomized, controlled trial. *Osteoporos Int* .2007;18(4) : 10.1007/s00198-006-0256-1 453-62 10.1007/s00198-006-0256-1 17103296

[5] : Effects of dynamic resistance exercise on bone mineral density in postmenopausal women: a systematic review and meta-analysis with special emphasis on exercise parameters. *Osteoporos Int* .2020;31(8) : 10.1007/s00198-020-05441-w 1427-1444 10.1007/s00198-020-05441-w 32399891 PMC7360540

# Response

**Published:** 2023-02-28

**Authors:** Rizky Suganda Prawiradilaga

**Introduction**

**P1:** We are sorry if we were mistaken. We got it from the Facts and Statistics International Osteoporosis Foundation (IOF) [cited 2017 Oct 4]. Some other references that said the same that 200 million women: COLOSTEO (the project has received funding from the European Union’s Horizon 2020 research and innovation programme under grant agreement No. 778277) ^1^, and de Villiers and Goldstein (2022) ^2^.

**P2:** Thank you. We have changed the reference to: Zhang S, Huang X, Zhao X, Li B, Cai Y, Liang X, *et al.* Effect of exercise on bone mineral density among patients with osteoporosis and osteopenia: A systematic review and network meta-analysis. *J Clin Nurs*. 2022 Aug; **31**(15–16):2100–11.

**P3 & 4**: We agree with you, we have changed a little bit “it is hypothesized in the present study that evidence-based multimodal training as health promotion will have an osteogenic effect” into “it is hypothesized in the present study that training (which combined evidence-based exercise) as health promotion will have an osteogenic effect”. We have revised it. 

**P4:** We have added the references:

- Watson SL, Weeks BK, Weis LJ, *et al.*:High-Intensity Resistance and Impact Training Improves Bone Mineral Density and Physical Function in Postmenopausal Women With Osteopenia and Osteoporosis: The LIFTMOR Randomized Controlled Trial. *J Bone Miner Res.* 2018 Feb; **33**(2):211–220. PubMed Abstract | Publisher Full Text
- Kistler-Fischbacher M, Yong JS, Weeks BK, *et al.*:A Comparison of Bone-Targeted Exercise With and Without Antiresorptive Bone Medication to Reduce Indices of Fracture Risk in Postmenopausal Women With Low Bone Mass: The MEDEX-OP Randomized Controlled Trial. *J Bone Miner Res.* 2021; **36**(9):1680–1693. Publisher Full Text
- Kistler-Fischbacher M, Weeks BK, Beck BR:The effect of exercise intensity on bone in postmenopausal women (part 2): A meta-analysis. *Bone.* 2021 Feb 1; **143**: 115697. Publisher Full Text
- Karinkanta S, Heinonen A, Sievänen H, *et al.*: A multi-component exercise regimen to prevent functional decline and bone fragility in home-dwelling elderly women: randomized, controlled trial. *Osteoporos Int.* 2007 Apr; **18**(4): 453–462. PubMed Abstract | Publisher Full Text
- Shojaa M, von Stengel S , Kohl M, *et al.*:Effects of dynamic resistance exercise on bone mineral density in postmenopausal women: a systematic review and meta-analysis with special emphasis on exercise parameters. *Osteoporos Int.* 2020 Aug; **31**(8):1427–1444. PubMed Abstract | Publisher Full Text | Free Full Text

**P5: **Thank you for the suggestion, We have added attendance rate as a feasibility endpoint in the Methods section. The feasibility outcome is already in the Results section.

**Methods**

**General:** We have clarified which outcomes were primary and which ones were secondary.

**P1:** It was explained in Participants section in then Methods section. The sedentary control group was from a previous study (which hasn’t been published) not a long time before the present study. The recruitment process itself was the same, via advertisement online and a local newspaper.

**P1:** Yes, we understand. The bone turnover occurs around 5 to 6 months, and we can detect the changes around that time of intervention. However, our study regarding the bone turnover marker (Prawiradilaga *et al. *(2020) ^3^) showed that acute exercise has an acute response on bone marker (P1NP). Regarding that, we hope that we can see the change in bone turnover markers as well as the bone mineral density in only 19 months. Why only 19 months? It was the feasible range of time we can manage before everyone went to summer vacation.

**P3: **Alright, thank you. We have deleted that part.

**P4:** Sedentary is defined as someone who didn’t engage in regular and systemic weight-bearing training or strength training in the last 2 years. We have added a note to distinguish active from sedentary.

- No minimum age, only maximum 70.
- 2 years post menopause
- Minimum at least 1 time a week, has a goal of the training.

**P5:** We have considered your suggestion and have moved the number of participants and drop outs to the Results section.

**P5: **Thank you.

**P8:** The progressive resistance training (PRT) consisted primarily of exercises using own bodyweight, with occasional use of medicine balls, resistance bands and sandbags (5–15 kg), starting with 15–20RM and slowly progressing to 6–12RM, with 2–3 sets. We have added this in the manuscript.

**P9: **We don’t think we have the information on short-term of BMD and body composition. We only measure the bone turnover markers for the short-term.

**P12/13:** They did not want to do the test.

**P14:** Thank you for the suggestion.

**Results**

**P1: **Our analyses were based on the delta values.

**P4: **Thank you for your suggestion. We have removed “However, LS (1.2%) and right total femur (0.3%) tended to increase (both, p < 0.1) for MMT”.

**Table 2: **We think it is good information to give the reader the mean femur BMD.

**Table 2/3: **There is significant difference between MMT and Control (CON) group. While the whole-body BMC showed decrease in CON group, the whole BMC in MMT group doesn’t change. It showed that the training might inhibit the whole-body BMC to decrease in postmenopausal women. The whole-body BMD was not increased, however, the right trochanter BMD in MMT was increased compared to CON.

**Discussion**

 Thank you for the suggestion, We have added regarding the recent studies which have shown that exercise intensity is positively associated with bone changes, and that low-intensity exercise is ineffective.

 Thank you for your suggestions, we have addressed other limitations.

1. The sample size was determined by the Author judgement based on the previous study on Bone Turnover Markers (Prawiradilaga *et al. *(2020) ^3^.
2. The posteriori power calculations were performed with Power and Sample Size Calculations version 3.0 for the paired t-test and performed with SPSS for the repeated ANOVA measurement. The results are in the link in the ‘Extended data’ section.

 

- Is the work clearly and accurately presented and does it cite the current literature?

We have cited the current literature

- Is the study design appropriate and is the work technically sound?

We have added, “This is a feasibility study in a local community with a non-randomized controlled trial approach.”

- Are sufficient details of methods and analysis provided to allow replication by others?

We think it is sufficient since the study method and the training program were described clearly.

**References**

1. Osteoporosis Facts [Internet]. COLOSTEO. [cited 2022 Aug 12]. Available online (date last accessed: 10/03/2023)
2. de Villiers TJ, Goldstein SR: Bone health 2022: an update. *Climacteric.* 2022 Jan 2; **25**(1):1–3. Publisher Full Article
3. Prawiradilaga R, Madsen A, Jørgensen N, *et al.*: Acute response of biochemical bone turnover markers and the associated ground reaction forces to high-impact exercise in postmenopausal women. *Biol Sport.* 2020; **37**(1): 41–48. PubMed Abstract
